# Rates of Evolution Differ Between Cell Types Identified by Single‐Cell RNAseq in Threespine Stickleback

**DOI:** 10.1111/mec.70565

**Published:** 2026-09-28

**Authors:** Maria L. Rodgers, Swapna Subramanian, Lauren E. Fuess, Wan He, Samuel V. Scarpino, Andrea J. Roth‐Monzón, Daniel L. Jeffries, Martine Seignon, Kathryn Milligan‐McClellan, Rebecca Carrier, Natalie C. Steinel, Daniel I. Bolnick

**Affiliations:** ^1^ Department of Ecology and Evolutionary Biology University of Connecticut Storrs Connecticut USA; ^2^ Department of Biological Sciences, Center for Marine Sciences and Technology North Carolina State University Morehead City North Carolina USA; ^3^ Department of Biology Texas State University San Marcos Texas USA; ^4^ Northeastern University, Institute of Experiential Artificial Intelligence Boston Massachusetts USA; ^5^ Division of Evolutionary Ecology, Institute of Ecology and Evolution University of Bern Bern Switzerland; ^6^ The Jackson Laboratory for Genomic Medicine Farmington Connecticut USA; ^7^ Department of Molecular and Cell Biology University of Connecticut Storrs Connecticut USA; ^8^ Department of Chemical Engineering Northeastern University Boston Massachusetts USA; ^9^ Department of Biological Sciences University of Massachusetts Lowell Lowell Massachusetts USA

**Keywords:** evolutionary rates, *Gasterosteus aculeatus*, immunity, single‐cell sequencing

## Abstract

Rates of evolutionary change vary by gene. While some broad gene categories are highly conserved with little divergence over time, others undergo continuous selection pressure and are highly divergent among populations or species. But such comparative evolutionary studies rely on gene ontology categories that are rarely validated in the study's focal species. An alternative is to determine species‐specific gene functions using single‐cell RNA sequencing (scRNAseq) that identifies cell types and the characteristic genes that each expresses. Here, we combine scRNAseq with evolutionary genomics to understand whether certain cell types exhibit faster evolutionary divergence (using their characteristic genes) than other types of cells (using intestine, head kidney, liver and gill tissues from an emerging model organism, the threespine stickleback). Merging scRNAseq with population genomic data, we show that cell types differ in the rate at which their characteristic genes evolve, as measured by allele frequency divergence among many populations (*F*_ST_) and the longer‐term effect of selection driving sequence differences between species (*dN*/*dS* ratios). Neutrophils, B cells and fibroblasts exhibit elevated *F*_ST_ at characteristic genes, while eosinophils in the intestine and thrombocytes in the head kidney exhibit lower *F*_ST_ than the average for 1000 random genes (*dN*/*dS* showed similar results). Genes' positions within the scRNAseq coexpression network (measured by graph theory metrics of centrality) also differed between cell types and there was a weak tendency for genes with higher closeness centrality to have higher *F*_ST_. These results highlight the value of merging scRNAseq technology with evolutionary population genomic data and reveal that genes which define immune cell types exhibit especially rapid evolution.

## 1 Introduction

Comparative evolutionary genetics studies have clearly established that the rate of evolutionary change varies between genes (Fay et al. 2001; Hahn 2008; Gout et al. 2010). Some categories of genes are highly conserved and exhibit very slow evolutionary divergence between populations or species, indicating negative selection purging new mutations. Other genes exhibit a signature of persistent positive selection favouring new mutations. For example, divergence among human populations is, on average, especially rapid for immune genes, particularly those involved in antihelminth immunity (Fumagalli et al. 2011; Vinkler et al. 2023; Flanagan et al. 2025). However, such studies tend to use coarse gene ontology (GO) categorizations to classify genes, which may not effectively reflect the function of or relations between, genes. This limitation is especially acute for non‐model organisms whose gene ontologies are inferred from very distantly‐related models. An alternative approach would be to fuse the relatively new technology of single‐cell RNA sequencing (scRNAseq) with evolutionary genomics (Carmona et al. 2017; Marioni and Arendt 2017; Mah and Dunn 2024; Tanay and Sebé‐Pedrós 2021). In non‐model organisms, scRNAseq can help identify cell types within tissue samples. These cell type identities can be used to infer the functions of the genes whose expression is enriched in those cells. This approach provides an alternative to GO classifications for non‐model organisms. One can then use these functional classifications to compare the evolutionary dynamics of genes expressed within different cell types, with different biological functions.

Here, we use scRNAseq to infer cell types in multiple tissues from threespine stickleback ( *Gasterosteus aculeatus* ), an emerging model organism in evolutionary genetics (Reid et al. 2021; McKinnon and Rundle 2002; Bell and Foster 1994; Cresko et al. 2006; Beck et al. 2022; Jones et al. 2012; Fuess and Bolnick 2023). We identified clusters of cells with similar transcriptome profiles and sets of genes whose expression is diagnostic for each cluster (cell type associated genes [Cell‐TAGs]). We then used a large population genomic dataset to identify the extent of among‐population allele frequency divergence at each of the Cell‐TAG loci. We also measure signatures of selection in between‐species divergence (*dN*/*dS*). We use these estimates to test whether the Cell‐TAGs from different types of cells exhibit different evolutionary rates on average. As a baseline for reference, we also evaluate evolutionary rates for a set of randomly chosen loci that are not cell‐type‐specific. We show that the rate of Cell‐TAGs evolution varies among cell types and is faster for some cell types involved in immunological processes. This variation in evolutionary rates is in part associated with cell and gene differences in gene co‐expression network architecture.

## 2 Methods

### 2.1 scRNAseq

Single‐cell libraries were generated from the intestines of three individual laboratory‐reared threespine stickleback, bred from parents caught in the Sayward Estuary in British Columbia, Canada. For head kidneys, single‐cell libraries were generated from nine total laboratory‐reared threespine stickleback (three each bred from Sayward Estuary, Roberts Lake and Gosling Lake, on Vancouver Island). All fish were collected in accordance with the Ministry of Forests, Lands and Natural Resource Operations of British Columbia (Scientific Fish Collection permits NA12‐77018 and NA12‐84188). To obtain these embryos, we trapped gravid female fish from each source population. Eggs were stripped from females and mixed with sperm from males' macerated testes. Fertilized eggs were transported to the University of Texas at Austin where fish were raised for 2 years before being transferred to the University of Connecticut (IACUC approval # A21‐025).

To generate cell suspensions, stickleback were euthanized with MS‐222 and intestines, livers, gills or head kidneys were removed. The head kidneys were from different individual fish, having been sampled a year earlier (first published in Fuess and Bolnick 2023) and re‐analysed here. For consistency, we used the same methods as Fuess and Bolnick (2023) for the intestine, liver and gill in this study. For intestine, liver and gills, three adult fish were used. For head kidneys, nine adult fish were used. Upon removal, intestines were initially cut into two pieces (each representing half of the total length of the intestine; these pieces were marked as the anterior and posterior intestine). The livers, gill and head kidneys were left intact. Each organ was placed into a sterile 24‐well plate on ice with 2 mL on R‐90 media (90% (v/v) RPMI 1640 (+l‐glutamine, l‐Phenol red; Gibco) + 10% (v/v) distilled water) in each well. From there, each piece of the intestine was cut longitudinally and then sliced into small fragments; head kidneys, gills and livers remained intact. The intestinal pieces and whole livers, gills and head kidneys were dissociated physically by using a sterile pipette tip. Liquid from each well of the plate was then individually passed through a 40‐μm nylon screen (BD Falcon) and 2 mL of chilled R‐90 was added to the resulting cell suspension. The suspension was spun for 10 min at 4°C and 550 *g*. Supernatant was discarded from each tube and cells were resuspended in 2 mL of chilled R‐90. Once again, the suspension was spun for 10 min at 4°C and 550 *g*. Supernatant was discarded from each tube and cells were resuspended in 1 mL of chilled R‐90. Cells were then transferred on ice to The Jackson Laboratory for Genomic Medicine in Farmington, CT for sequencing.

Once cells were transferred to The Jackson Laboratory for Genomic Medicine (within 1 h of resuspension), cells were washed and suspended in PBS containing 0.04% BSA and immediately processed as follows. Cell viability was assessed on a Countess II automated cell counter (ThermoFisher) and an estimated 12,000 cells were loaded onto one lane of a 10× Genomics Chromium Controller. Single cell capture, barcoding and single‐indexed library preparation were performed using the 10× Genomics 3′ Gene Expression platform, v3.1 chemistry and according to the manufacturer's protocol (#CG000204) (Zheng et al. 2017). cDNA and libraries were checked for quality on Agilent 4200 Tapestation, quantified by KAPA qPCR and sequenced on an Illumina NovaSeq 6000 targeting 6000 barcoded cells with an average sequencing depth of 50,000 read pairs per cell (approximately 13% of an S4 flow cell).

### 2.2 scRNA‐Seq Data Processing and Analysis

Illumina base call files for all libraries were converted to FASTQs using bcl2fastq v2.20.0.422 (Illumina) and FASTQ files were aligned to reference genome constructed from the 2020v5 *G. aculeatus* assembly and annotation files available at https://stickleback.genetics.uga.edu/ (Nath et al. 2021). Briefly, annotations from Ensembl (release 95) were combined with repeat, Y chromosome and revised annotations from Nath et al. using AGAT (0.4.0) and a STAR‐compatible reference genome was generated Cell Ranger (version 6.0.0, 10× Genomics) using these annotations and the v5 assembly from Nath et al. (2021; Dainat 2022). The Cell Ranger count pipeline was used to construct a cell‐by‐gene counts matrix for each library, subsequently analysed using Scanpy 1.3.7 and the Loupe Cell Browser (10× Genomics) (Wolf et al. 2018).

Each counts matrix was individually subjected to quality control filtering, such that cells with fewer than 400 genes, more than 20% mtRNA content and more than 500 haemoglobin transcripts were discarded from downstream analysis. Additionally, cells with more than 12,500, 15,000 and 25,000 UMI counts were discarded from libraries DB21009, DB2012 and DB21007, 008, 010, 011, respectively. Filtered counts matrices were concatenated, normalized by per‐cell library size and log transformed. The expression profiles of each cell at the 3500 most highly variable genes (as measured by dispersion Ranwez et al. 2021; Satija et al. 2015) were used for principal component (PC) analysis and subsequently batch corrected using Harmony (Korsunsky et al. 2019). The batch corrected PCs were utilized for neighbourhood graph generation (using 40 nearest‐neighbours) and dimensionality reduction with UMAP (McInnes et al. 2020). Clustering was performed on this neighbourhood graph using the Leiden community detection algorithm (Traag et al. 2019). Subclustering was performed on a per‐cluster ad hoc basis to separate visually distinct subpopulations of cells. This UMAP embedding and clustering metadata were then imported into the Loupe Cell Browser (using Cell Ranger aggr, version 6.0.0) for interactive analysis.

Upon importing UMAP embedding and clustering metadata into the Loupe Cell Browser, we then followed steps taken by Fuess and Bolnick (2023) to identify marker genes. Briefly, we generated lists of Cell‐TAGs using the ‘Globally Distinguishing’ feature and classified marker genes as genes upregulated in each cluster (compared with all other cells) with an adjusted *p* < 0.10. Once we had a list for each cluster, we then examined the literature to identify the functions of Cell‐TAGs as documented in other species (e.g., mice, zebrafish, etc.). We used the descriptions of multiple Cell‐TAGs per cell cluster to decide how to categorize it as a cell type.

### 2.3 poolSeq to Estimate Population Genetic Divergence

We used previously published poolSeq data (Weber et al. 2022), which provided estimates of allele frequencies in stickleback populations from 23 lakes (15 in British Columbia, 8 in Alaska) and 3 marine sites (2 in British Columbia, 1 in Alaska). For each population, 100 fish were sampled by minnow traps to obtain fin clips with permits from local governments (BC: NA07‐32612; AK: SF2019‐085) and university IACUCs (UT Austin: 07‐032201; UConn: A19‐015). The number of individuals included in each population pool is in Table S1. DNA was extracted with magnetic beads, following the Bio‐One‐Magnetic‐Beads protocol (Oberacker et al. 2019) with some modifications. Modifications included different lysis buffer (10 mM Tris pH 8.3, 50 mM KCl, 1.5 mM MgCl2, 0.3% Tween‐20 and 0.3% NP‐40). We added 255 μL of the lysis buffer and 20 μL of Proteinase K per sample, left overnight at 56°C. We also shaked the plate for 6 min with the isopropanol, added 120 ul of beads and added 300 μL of isopropanol for the first wash. We did three 80% ethanol washes, all as indicated in the protocol. For the final elution, we shook the plate for 7 min. We used modified Serapure beads for the extractions. We quantified DNA extract concentrations using Picogreen on a Tecan‐Magellan plate fluorometer and used these concentrations to pool equimolar amounts of DNA from each of 100 fish per population to generate one DNA pool per population. These pools were subjected to whole genome sequencing at a target depth of 200× (e.g., 1× per allelic copy), using an Illumina NovaSeq 6000 at the Center for Genome Innovation in the University of Connecticut. To supplement our poolSeq data, we added published poolSeq data (NCBI SRA database accession PRJNA671824) (Aguirre et al. 2022) from an additional Alaskan marine population (Rabbit Slough) and three additional Alaskan lake populations recently founded by marine fish (Kingman et al. 2021).

The raw sequencing reads for the 24 newly sequenced population are deposited in NCBI SRA Bioproject PRJNA850376. The populations were sequenced to an average coverage of 100–150, which is sufficient to allow an accurate estimation of allele frequencies. We used the pp_align programme in the PoolParty pipeline to process the raw reads and to call SNP variants (Micheletti and Narum 2018). The parameter settings we used in pp_align were BQUAL = 20, MAPQ = 10, SNPQ = 20, MINLENGTH = 25, INWIN = 2, MAF = 0.00005, MINDP = 10. Then, we used the R package poolfstat to calculate the pairwise unbiased Weir‐Cockerham *F*_ST_ values for each SNP, for each pairwise population comparison. These calculations used the following settings: min.rc = 4, min.cov.per.pool = 10, max.cov.per.pool = 2000, nlines.per.readblock = 1e + 06,noindel = T (Hivert et al. 2018).

For the purpose of this study, we chose to focus on population genetic divergence arising from marine stickleback's colonization of freshwater lakes, following Pleistocene deglaciation (Jones et al. 2012). We therefore focus on *F*_ST_ values between each naturally established lake population and the nearby marine population that likely represents the genotypes of the ancestral fish that would have colonized local freshwater habitats (Laine et al. 2024). For each SNP, we used the R package matrixStats to calculate the average marine‐to‐lake *F*st, using Rabbit Slough as the marine reference for Alaskan lakes and Sayward Estuary as the marine reference for Vancouver Island lakes. We omitted human‐introduced populations from these calculations. We then used the R package dplyr to filter the SNPs and associated *F*_ST_ data by the base pair locations, to focus on SNPs within or around the Cell‐TAG genes identified through scRNA, including SNPs within 5 kb on either side of a given locus (Wickham et al. 2021). For comparison, 1000 random genes were sampled from all stickleback genes using the R function sample and dplyr was used to filter the SNPs and associated *F*_ST_ data by the base pair locations of the random genes with 5 k base pairs added on either side. We averaged the *F*_ST_ estimates for many SNPs within each gene (and its flanking 5 kb) to obtain a gene‐level average *F*_ST_.

We then tested for among‐cell type differences in evolutionary divergence. Each cell type was represented by multiple Cell‐TAGs, each with a gene level mean *F*_ST_. We treat the different Cell‐TAG genes as replicates within a given cell type and conducted ANOVAs to test for among‐cell‐type differences in gene mean *F*_ST_. We did this separately for each tissue type.

### 2.4 Microevolutionary Divergence

Next, we estimated rates of microevolutionary divergence (*F*_ST_) in stickleback using whole genome sequences from 28 stickleback populations (2 marine populations as an outgroup and from various‐sized lakes in Alaska (*N* = 8)) and on Vancouver Island (*N* = 15 lakes). From each population, we pooled ~100 individuals' DNA for PoolSeq to obtain genome‐wide allele frequency estimates from each population (see Weber et al. 2022 for details and population information). We then calculated pairwise *F*_ST_ to measure between‐population allele frequency differences at each SNP in the genomic dataset, for all pairwise comparisons between populations (the average gene in our dataset had 211.1 SNPs within its window, range of 64–3130, standard deviation of 336.5). A neighbour‐joining phylogenetic tree based on all pairwise *F*_ST_ estimates (redrawn from Weber et al. 2022) confirms substantial population divergence among all lakes and between marine and freshwater populations (Figure 2a), with Alaskan and Vancouver Island lakes forming distinct clades and within‐region genetic structure corresponding closely to different watersheds and sub‐regions. For present purposes, we focus on selection associated with the dramatic ecological shift when marine stickleback colonized freshwater habitats. We focus on pairwise *F*_ST_ between an Alaskan anadromous marine population (Rabbit Slough) and 8 native Alaskan lake populations (excluding artificially founded lake populations) and *F*_ST_ between a Vancouver Island anadromous marine population (Sayward Estuary) and 13 Vancouver Island lake populations (excluding two lakes suspected of being recent marine invasions after a tsunami; Frederick and Pachena Lakes). For each SNP, we calculated the Weir–Cockerham *F*_ST_ between each lake population and its nearest marine counterpart (Rabbit Slough for Alaskan lakes; Sayward Estuary for Vancouver Island), resulting in 21 estimates per SNP. Then we averaged these 21 lake‐marine *F*_ST_ estimates to obtain one SNP‐level mean *F*_ST_. Figure S1 presents a comparison of *F*_ST_ values in Alaska versus Vancouver Island for individual SNPs; Figure S2 presents the density and frequency distribution of these SNP‐level mean *F*_ST_ values for a marine versus freshwater comparison. Next, we averaged these SNP‐level mean *F*_ST_ values across all SNPs within a given gene (including introns and a 5 kb window on either side) to obtain a gene‐level mean *F*_ST_ of among‐population divergence (Figure S2).

Using nested linear mixed models, we tested whether SNP *F*_ST_ values depended on the Cell‐TAGs gene they are close to, with SNPs as the level of replication within genes, which are nested within cell types (linear model specification: *F*_ST_ ~ intercept + cell_type|gene, with normal residuals; details can be found in the R scripts in the data archive accompanying this paper).

### 2.5 Phylogenetic Branch Lengths and *dN*/*dS* Calculations

To evaluate evolutionary rates over longer time scales, we also measured molecular genetic divergence among stickleback species, whose evolution spans millions of years. We first filtered the latest *G. aculeatus* genome annotation (NCBI RefSeq accession: GCA_016920845.1) (Nath et al. 2021) to remove all but the longest isoform for each gene using AGAT v1.0 (Dainat 2022). The remaining coding sequences were used to identify one‐to‐one orthologs (using Orthofinder v2.5.4) in the assemblies of three other Gasterosteiformes with whole genome species: *G. wheatlandi* (unpublished), *Apeltes quadracus* and *Aulorhynchus flavidus (*Emms and Kelly 2019; Li et al. 2022). Each gene alignment was trimmed using MACSE v2.07 and TrimAl v1.4.1 to remove non‐homologous regions and regions with little data across the four species (Ranwez et al. 2021; Capella‐Gutiérrez et al. 2009). Trimmed gene alignments were then used to calculate *dN*/*dS* and tree length (all sites) using an M0 model in the CODEML programme in PAML v4.9 (Yang 2007) based on the underlying tree topology ( *A. flavidus* ( *A. quadracus* ( *G. wheatlandi* , *G. aculeatus* ))) taken from Liu et al. (2022). We then used an ANOVA to test whether Cell‐TAGs' *dN*/*dS* or relative branch lengths were different from those of randomly selected control genes. And we used an ANOVA to test whether these molecular evolution rates differed between the Cell‐TAGs for different cell types identified by scRNAseq.

### 2.6 Network Analysis

In addition to inferring cell types, scRNAseq can provide estimates of among‐cell covariation in gene expression to infer cell‐level co‐expression networks. We used our scRNAseq to test whether genes' positions within this network could also impact rates of evolution. There were six sets of intestine scRNA‐seq data used for generating single‐cell gene co‐expression networks. These datasets consist of three anterior intestine scRNA‐seq datasets (AI1, AI2, AI3) and three posterior intestine scRNA‐seq datasets (PI1, PI3, PI4). For each scRNA‐seq dataset, we generated a gene co‐expression network by calculating pairwise Spearman's rank correlation coefficient among the gene pairs. Fully connected weighted co‐expression networks were used for network feature extraction. Cells and genes with close‐to‐zero variation were filtered out. Specifically, for specimens AI1, AI2, AI3, PI1, PI3 and PI4 (AI = anterior intestine; PI = posterior intestine), 20%, 10%, 0%, 10%, 10% and 10% of the cells, respectively and 36%, 36%, 46%, 36%, 47% and 35% of the genes with the least variance after scaling were removed. Nodes in these networks represent genes and the edge weight between any pair of two genes represents the similarity of their expression profile across cells in the intestine single‐cell dataset, averaged across biological replicates from different fish. For each gene, we considered four standard graph theory measures of a gene's connectivity within a network: degree centrality, eigenvector centrality, Katz centrality and closeness centrality. Degree centrality counts the number of nodes (other genes) that each focal gene is immediately associated with. Eigenvector centrality weights those connections by how highly connected those other genes are. Katz centrality does the same but gives less weight to more distant nodes. Closeness centrality measures how far one must travel through the network, on average, to connect a focal gene to all other genes. We then used linear models to test whether each of these network features were correlated with rates of evolution (*F*_ST_) or differed between cell types.

## 3 Results

### 3.1 Cells and Reads Per Cell Obtained From scRNAseq

We obtained scRNAseq data from anterior intestines, posterior intestines, liver and gills (*N* = 3 fish each [*note:* the same three fish were used for all organs]) and head kidneys (a.k.a. pronephros, nine fish total). Libraries for intestinal tissues ranged from 12,278 to 30,789 estimated cells (mean reads per cell ranged from 13,173 to 35,513), head kidneys ranged from 8161 to 19,382 estimated cells (mean reads per cell ranged from 15,580 to 55,204), livers ranged from 8436 to 9662 estimated cells (mean reads per cell ranged from 32,088 to 52,690) and for gills estimated cells ranged from 7327 to 9089 (mean reads per cell ranged from 41,759 to 65,325). For intestines, median genes per cell ranged from 257 to 779, in the head kidneys, median genes per cell ranged from 307 to 707, in livers median genes per cell ranged from 309 to 372 and in gills median genes per cell ranged from 327 to 380.

### 3.2 UMAP and t‐SNE Clustering

Uniform Manifold Approximation and Projection (UMAP) and t‐Distributed Stochastic Neighbour Embedding (t‐SNE) clustering analyses identified 23 clusters of cells for the intestine (24 in the head kidney, 4 in the liver, 5 in the gills). Fuess et al. previously characterized the cell type identity of nine distinct cell clusters in the stickleback head kidney, of the 24 total clusters estimated by t‐SNE (Fuess and Bolnick 2023). With our new data from intestinal samples, we were able to draw a correspondence between 11 of the intestinal clusters in our study and cell types as defined in previous studies, by matching cluster‐specific marker genes based on the identity of genes with elevated expression within a given cluster (Figure 1). Each cell cluster was diagnosed by high expression of at least one gene (typically multiple genes), which we refer to as cell‐type specific genes (Cell‐TAGs). Some examples of Cell‐TAGs from the intestine are plotted in Figure 1. For instance, *cel* is a gene expressed at high frequencies only in cell cluster 14 and in humans is transcribed in the pancreas and in the mammary gland during lactation, allowing us to define this cell cluster as acinar cells (Lidberg et al. 1992). Cluster 17 is identified as intestinal epithelial cells based on strong expression of several genes, including *spdef* and *agr2*, two genes strongly associated with goblet cells (a specialized type of epithelial cell) (McCauley and Guasch 2015). The 11 identified clusters in the intestine are: haematopoietic cells, stem cells, eosinophils, acinar cells, intestinal epithelial cells, erythrocytes, proerythroblasts, enterocytes, neutrophils, myeloblasts and monocytes (Table S2). For several of these cell types, histological and RNA‐Scope analyses of intestinal sections were able to validate these scRNAseq based inferences (Pahiar et al. 2026). All clusters in the liver and gills were identifiable to known cell types based on the identity of their differentially expressed genes. The four identified clusters in the liver are: neutrophils, erythrocytes, hepatocytes and Kupffer cells (Table S3). The five identified clusters in the gill are erythrocytes, neutrophils, natural killer cells, B cells and epithelial cells (Table S4). As the intestine showed a higher diversity of cell types than the liver and gills, we elected to primarily focus on the intestine, but also report corresponding results from other tissues.

**FIGURE 1:**
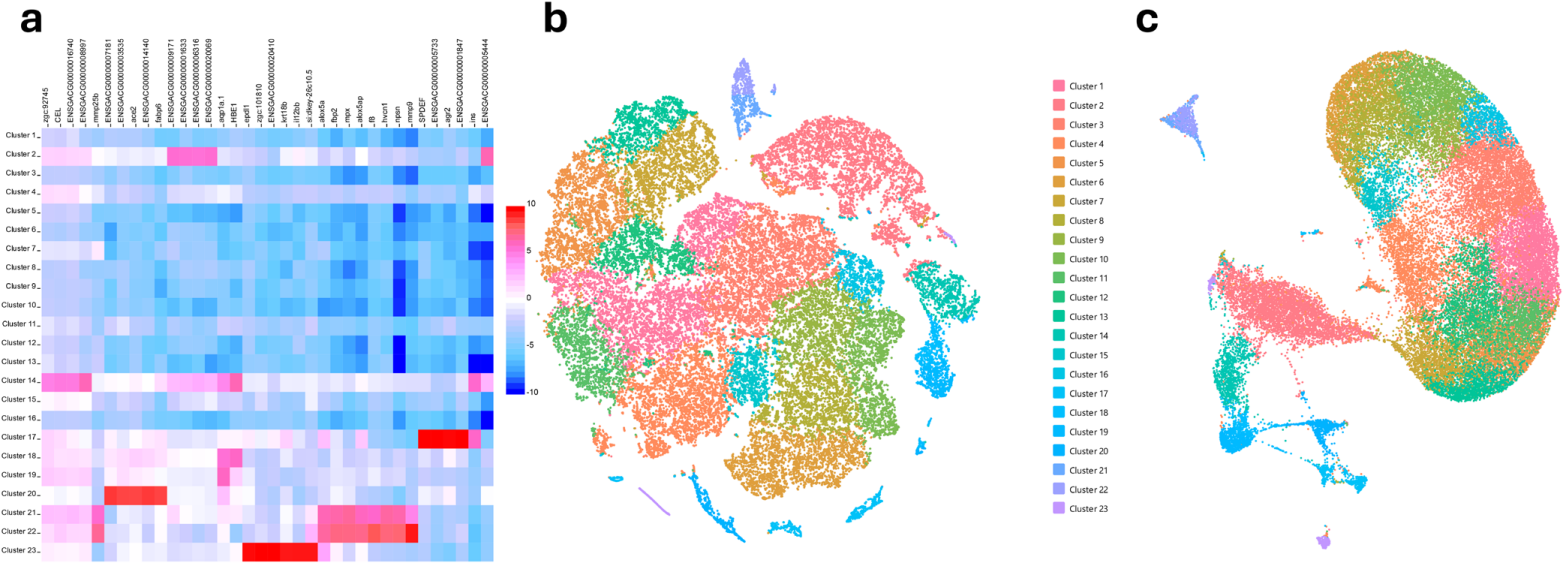
(a) Heatmap of the intestinal cell clusters presented as upregulated genes per cluster, compared to the entire dataset. Rows are cell clusters and gene transcripts are columns (with either gene name if annotated in the reference genome or transcript ID). Red signifies highly expressed genes (as compared to the entire dataset) and blue signifies downregulated genes (as compared to the entire dataset). Red boxes represent genes whose expression is especially high in a particular cell cluster, thus identifying cell type‐specific genes (Cell‐TAGs) whose evolutionary rates we query. (b) t‐SNE projection and (c) UMAP projection of all intestinal gene clusters (the key of cluster numbers and their corresponding colours are between (b) and (c)). These individual figures were generated using Loupe Browser 7.

### 3.3 Microevolutionary Divergence

Table S5 provides the overall and regional values of gene‐level *F*_ST_ means, with standard deviations, ranges and the number of SNPs for a sample of top‐*F*_ST_ genes. Some loci exhibit fixed or nearly fixed differences in allele frequency indicative of strong divergent selection (Figure 2b). A genome‐wide scan of these averaged *F*_ST_ values (Figure 2c) confirms that there is among‐gene variation in the rate of evolutionary divergence, consistent with prior studies of marine to freshwater adaptation in stickleback (Jones et al. 2012).

**FIGURE 2:**
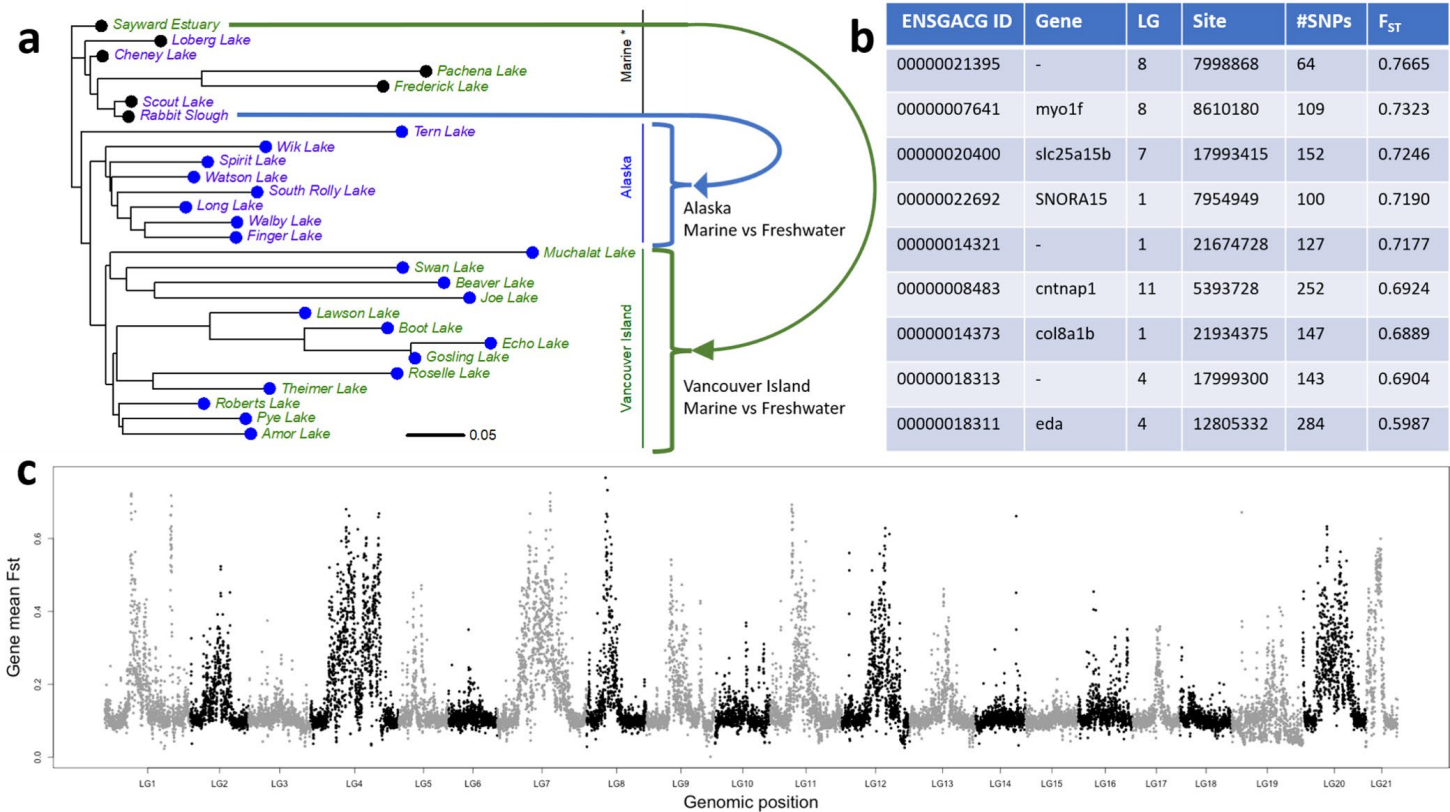
(a) A neighbour‐joining phylogenetic tree of 28 stickleback populations based on pairwise *F*_ST_ values for all Chromosome 15 SNPs identified with Poolseq (adapted from Weber et al. [27]). Freshwater populations are coloured blue, marine populations are coloured in black including three Alaskan lakes in which marine fish were introduced to lakes in the past few decades by humans (Loberg, Cheney and Scout) and two lakes on Vancouver Island where a historical tsunami is believed to have caused a replacement with marine fish (Frederick and Pachena). Alaskan lakes form a clade and Vancouver Island lakes form a clade (setting aside known or presumed marine introductions). We calculated marine to freshwater genetic divergence within each geographic region (Alaska marine to Alaska lakes, blue arrow; Vancouver Island marine to Vancouver Island lakes, green arrow), for each lake. We then averaged these *F*_ST_ values for each SNP, then averaged across SNPs within each gene (with flanking sequences). (b) A table of nine genes with the highest mean *F*_ST_, showing the ENSGACG gene ID, gene name (if known), linkage group, base position for the center of the gene, the number of SNPs within the gene and 5 kb flanking region and mean *F*_ST_ across the SNPs. The last row is *eda*, a well‐documented target of selection during marine to freshwater evolution (Capella‐Gutiérrez et al. 2009). (c) A Manhattan plot showing mean marine‐freshwater *F*_ST_ for each gene (points), colouring (grey vs. black) shows alternating linkage groups.

This among‐gene variation in the strength of divergent selection is, in part, attributable to the cell type within which the genes function. Merging the scRNAseq results with our population genomic data, we find that selection (e.g., exceptionally high *F*_ST_) is, on average, stronger for Cell‐TAGs associated with certain cell types, than for other cell types for both intestinal cell clusters (Figure 3a) and head kidney cell clusters (Figure 3b). Likelihood ratio comparisons of models with fixed effects of cell type versus just an intercept confirmed significant among‐cell‐type variation (log likelihood ratio LLR = 45.5, df = 17,4, *p* < 0.0001). Erythrocytes, for example, averaged *F*_ST_ = 0.106, compared to *F*_ST_ = 0.270 for B cells. The cell types with elevated *F*_ST_ at Cell‐TAGs include neutrophils, B cells and fibroblasts (nested mixed model ANOVA, LLR = 37.16, df = 11,4, *p* < 0.0001). We also contrasted Cell‐TAGs *F*_ST_ values against the gene‐level mean *F*_ST_ for 1000 randomly selected genes from the genome (Figures 3 and S2). For instance, fibroblasts have an average Cell‐TAGs *F*_ST_ = 0.185, which exceeds 80.5% of all random genes' *F*_ST_. Haematopoietic cells, APCs, red blood cells and natural killer cells were not significantly different from the 1000 random genes' *F*_ST_. Other cell types had significantly lower *F*_ST_ than the average for 1000 random genes, including eosinophils in the intestine and thrombocytes in the head kidney, suggesting that the Cell‐TAGs for these cells experience stabilizing selection that slows evolution. Note that natural killer cells had the highest gene‐level *F*_ST_ values, but as this cluster had few Cell‐TAGs (3 loci), we are not confident these represent exceptional selection at the cell level.

**FIGURE 3:**
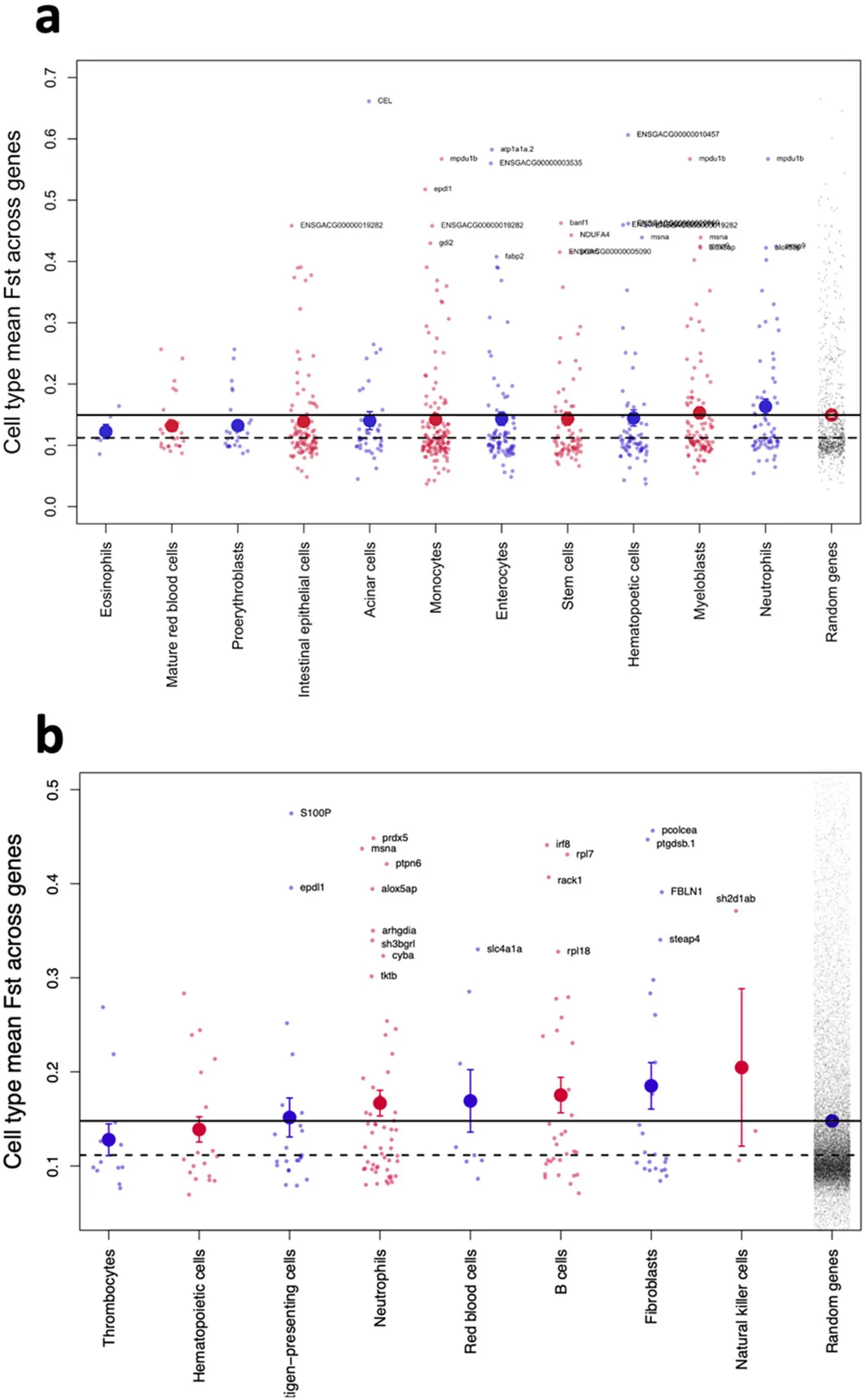
Evolutionary divergence between marine and freshwater stickleback (mean *F*_ST_) differs significantly between cell types as defined by (a) intestinal single‐cell RNAseq cell clusters and (b) head kidney scRNAseq cell clusters. Small data points are the cell‐type‐specific genes (Cell‐TAGs) that diagnose each cell type. Larger circles are averages across all Cell‐TAGs for a given cell type (with 1 standard error confidence intervals). Cell types are alternately coloured. At the far right of each plot are divergence measures for 1000 coding genes sampled randomly from the genome (tiny black points) with the mean (solid dot and solid horizontal reference line) and median (horizontal reference line) for comparison. For each panel the top Cell‐TAGs (highest mean *F*_ST_) are labelled with gene names. Number of Cell‐TAGs for each cell type in (a) eosinophils: 8, mature red blood cells: 37, proerythroblasts: 37, intestinal epithelial cells: 185, acinar cells: 84, monocytes: 193, enterocytes: 140, stem cells: 84, haematopoietic cells: 145, myeloblasts: 126, neutrophils: 103 and (b) thrombocytes: 17, haematopoietic cells: 35, antigen‐presenting cells: 61, neutrophils: 100, red blood cells: 20, B cells: 56, fibroblasts: 39, natural killer cells: 8.

### 3.4 Gene Network Connectivity

To evaluate whether among‐gene variation in evolutionary rate is influenced by gene network connectivity, we mapped loci included in this study onto a co‐expression network. Briefly, using scRNA transcriptomic data from both anterior intestines and posterior intestines we reconstructed a co‐expression network and calculated degree, eigenvector, Katz, betweenness and closeness centrality scores for each gene in the network (see Figure 4 legend for network statistic definitions). Using likelihood ratio comparisons of nested models with a fixed effect of immune‐gene‐status versus just an intercept we find significant variation in gene network centrality exists between immune‐ and non‐immune‐associated genes (LLR = 3.898, df = 1, *p* = 0.048). There is a significant positive correlation between closeness centrality and gene *F*_ST_ (Figure 4a). This means that evolutionary divergence is slightly faster for genes that are more strongly interconnected to most other genes, a phenomenon thought to imply greater potential for pleiotropic effects of genetic change. Additionally, we observed that gene network centralities varied among scRNA‐seq‐defined cell clusters (Figure 4b). However, the observed difference between immune‐ and non‐immune‐associated gene network centralities does not significantly explain the observed differences in *F*_ST_ (LLR = 0.410, df = 1, *p* = 0.522). Relaxing assumptions of linearity, we constructed a series of machine learning models—which allow for complex, non‐linear relationships—to predict *F*_ST_ from gene network centrality measures. Based on cross‐validation prediction performance, the machine learning models show at best a weak association between gene centrality and gene *F*_ST_ (Figure 4c). Taken together, our analyses show that—despite correlations existing between gene network features and *F*_ST_—the network features alone are inadequate to explain observed variation in evolutionary rate.

**FIGURE 4:**
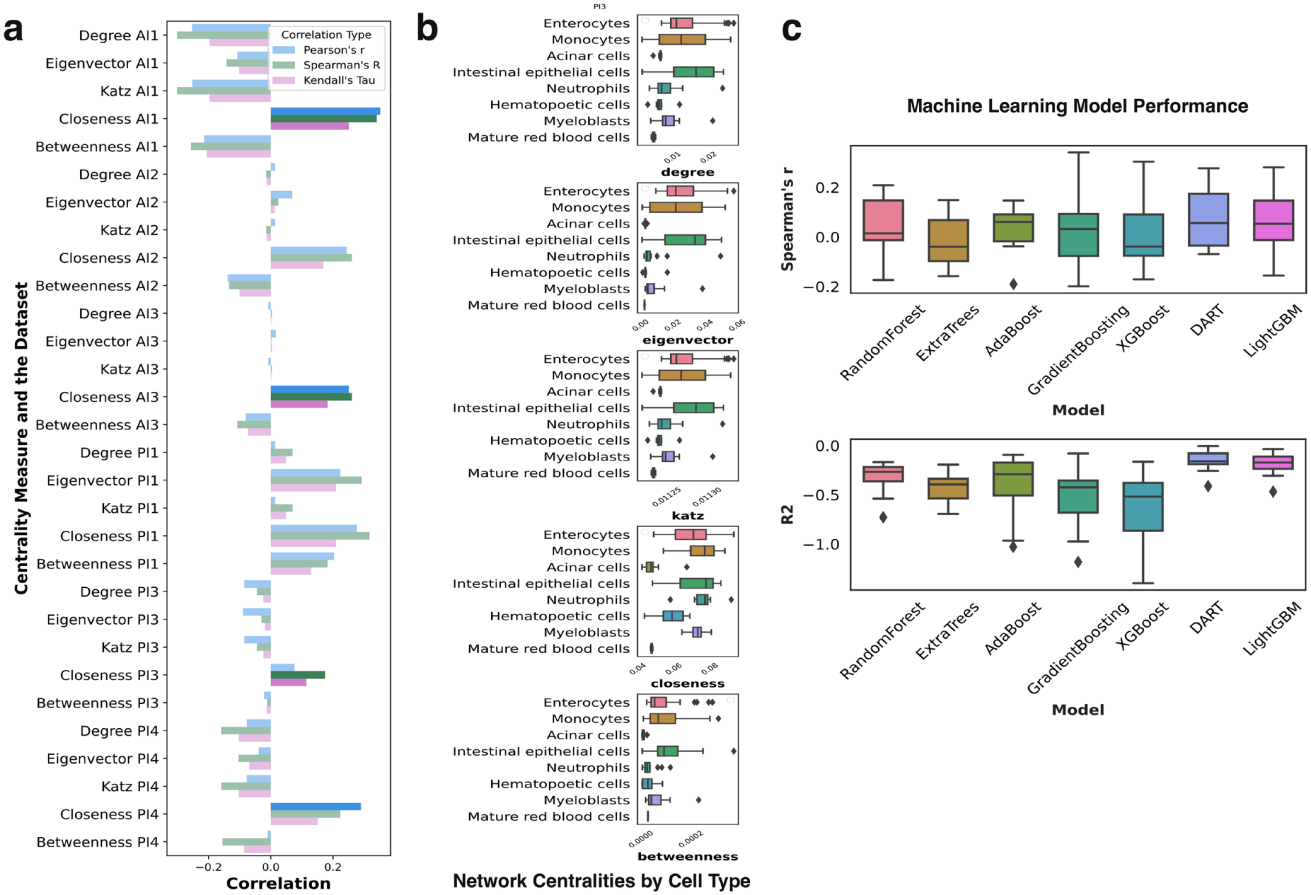
(a) Correlations between individual network features and gene evolutionary rates. Significant correlations with *p*‐values smaller than 0.05 were highlighted. Degree refers to the number of edges connecting a focal gene to other neighbouring genes in the network. Eigenvector centrality measures a nodes' importance based on both how many genes it is connected to and their own connection strength. Katz centrality measures the relative number of paths in the whole network that pass through a given gene. Closeness centrality measures the typical shortest paths from a focal gene to all other genes in the network. Betweenness centrality measures how often a focal gene lies on the shortest path between other genes. Each measure is calculated for the anterior and posterior intestines (AI or PI), separately for each sample (fish number 1, 2, 3 or 4). (b) Gene network centrality variations across cell types. (c) Model performance summary by *R* ^2^ score and Spearman's rank correlation coefficient between the predicted gene *F*_ST_ and the true *F*_ST_ values, obtained through fivefold cross‐validation.

### 3.5 Additional Metrics of Selection

Biologists often expect that rates of molecular evolution on short time scales can be extrapolated to longer time scales. However, these time scales can be decoupled. For instance, strong frequency‐dependent selection could lead to rapid fluctuations in allele frequencies over a few generations (and population divergence) that may not produce net genetic change over many generations. Therefore, in addition to our short‐term measure of selection (*F*_ST_), we calculated metrics of selection over longer time scales (phylogenetic branch lengths and *dN*/*dS*). We then tested whether *dN*/*dS* and tree length for Cell‐TAGs differed between cell types, within each of four tissues (intestine, head kidney, gill and liver).

The metric *dN*/*dS* measures the ratio of non‐synonymous substitutions that might be visible to selection, to synonymous substitutions that are largely expected to be neutral. Stabilizing selection tends to lead to a low *dN*/*dS* when new mutations that change amino acid sequences tend to be harmful. In general, *dN*/*dS* for Cell‐TAGs is less than 0.5, implying overall that selective constraints tend to remove amino‐acid‐changing mutations (Figure S3). However, this ratio varied significantly among cell types (Figure S3; liver *F*_10,188_ = 2.69, *p* = 0.0042; intestine *F*_13,94_ = 5.15, *p* = 0.0024; head kidney *F*_5,177_ = 3.027, *p* = 0.0120; gills *F*_4,57_ = 1.854, *p* = 0.1320). Within the liver, stem cells and eosinophils had the lowest *dN*/*dS*, whereas enterocytes, acinar cells and erythroblasts had the highest *dN*/*dS*. In head kidneys, haematopoietic stem cells and natural killer cells had lower *dN*/*dS* than platelets or neutrophils. In the liver, hepatocytes had higher *dN*/*dS* than neutrophils, which were notably low. This raises an interesting observation that the Cell‐TAGs for neutrophils in the liver are different from the Cell‐TAGs for neutrophils in the head kidney and the latter have higher *dN*/*dS*. Although there was no significant variation in *dN*/*dS* among cell types in gill samples, the trend is for natural killer cells and neutrophils to have lower *dN*/*dS* than B cells, epithelial cells or erythrocytes. Also notably, *dN*/*dS* tended in general to be higher for Cell‐TAGs in intestinal cells (of any type) relative to Cell‐TAGs in the head kidney cells (Figure S3). Figure S4 demonstrates variation in allele frequency divergence among Cell‐TAGs.

We obtain somewhat similar results when we focus on phylogenetic tree length per gene as a metric of genic substitution rates. Genes under stronger positive selection tend to accumulate more substitutions overall resulting in greater overall branch lengths, whereas selective constraints can compress branch lengths. Cell types differed in tree length for cells in the liver (*F*_3,94_ = 5.55, *p* = 0.0015) and head kidney (*F*_5,177_ = 2.314, *p* = 0.0457), but not gill or intestine (both *p* > 0.1), as illustrated in Figure S5. In the head kidney, neutrophils and platelets again had higher evolutionary rates than haematopoietic stem cells. In the liver, hepatocytes had higher rates of substitutions than neutrophils (which were again lower than for neutrophils Cell‐TAGs in the head kidney).

Tree length and *dN*/*dS* were positively correlated (*r* = 0.489, *t* = 7.68, df = 188, *p* < 0.0001). However, across cell types, on average these macroevolutionary metrics were not correlated with microevolutionary rates (among‐population *F*_ST_ versus *dN*/*dS r* = 0.317, *p* = 0.315; *F*_ST_ versus tree length *r* = −0.398, *p* = 0.200). This suggests the intriguing inference that the genes most quickly evolving between species are not the same as the genes most quickly diverging among populations within species.

## 4 Discussion

scRNAseq emerged in the past decade as an important tool in cell biology, systems biology, oncology and related fields (Li et al. 2021; Huang et al. 2023; Boxer et al. 2025; Paik et al. 2020; Potter 2018). However, this approach has yet to be widely adopted in evolutionary biology, in part because it remains too expensive to apply to the kinds of sample sizes required of population level studies. One way that scRNAseq can prove useful is by complementing the GO classification systems. GO terms are widely used to interpret the functional nature of population genomic signals of selection and transcriptomic divergence between populations. Given a list of genes with particularly strong selection (or differential expression), one can ask whether this list is enriched for particular kinds of biological functions, relative to null expectations. A widely acknowledged caveat about this approach is that genes' GO classifications are derived from a few model organisms, which are often distantly related to whichever non‐model species one chooses to study. Gene functions may change across such large expanses of evolutionary time. In contrast, scRNAseq can be readily applied to many non‐model species, to create a bespoke atlas of major cell clusters and the genes that are distinctively expressed within each cell cluster (cell‐type associated genes or Cell‐TAGs). As we illustrate here, Cell‐TAGs' clusters can be used, in place of GO, to investigate evolutionary questions about genome‐wide variation in rates of molecular divergence.

Rates of evolution (allele frequency change, substitution rates) are known to vary among genes within a genome (Gillespie 1992). Some loci are subject to strong constraints that eliminate new mutations, whereas other loci experience persistent positive selection favouring the replacement of ancestral alleles by new mutations. This variation in evolutionary rate can be attributed to various genetic forces (varying mutation rates or recombination rates) and differences in the strength of selection. Immune genes in particular have been observed to evolve quickly, ostensibly because the antagonistic ‘arms race’ between hosts and pathogens or parasites leads to a constantly changing selection pressure (Fumagalli et al. 2011; Vinkler et al. 2023; Flanagan et al. 2025). Analyses of these differences in rates of genic evolution are usually applied to genes individually, ignoring the joint action of genes. Or, analyses use coarsely defined GO derived from distant model species. A useful alternative that we explore here is to leverage data‐driven functional roles, obtained from the same study organism using single‐cell RNAseq data. The merger of scRNAseq data with population genomics allows gene function assignments (by cell cluster) within the study organism. We use this approach to show that the rate of genic evolution varies between scRNAseq‐defined cell clusters, within each of several organs: intestine, head kidney, liver and gills (Figure S5). In these tissues, the genes that distinguish cell clusters (cell‐TAGs) evolve at different rates (on average) depending on the cell type they are associated with. We are thus able to identify cell types whose function is likely to evolve especially quickly and conserved cell types. The faster evolving cell clusters are consistently central players in the adaptive and innate immune systems. This faster evolution of immune‐related genes is consistent with previous findings (Fumagalli et al. 2011). Immune defences are believed to evolve rapidly because they are perpetually engaged in antagonistic co‐evolution with parasites, which evolve counter‐adaptations to changing host defences (e.g., the evolutionary ‘Red Queen's Race’ model) (Van Valen 1973). It is important to note that our analyses focus on microevolutionary divergence between marine and freshwater stickleback and so different results might be obtained when studying adaptive divergence between other ecotype combinations. But, the marine to freshwater transition has been arguably the strongest force for rapid and parallel evolution in stickleback (Jones et al. 2012; Kingman et al. 2021), often driving evolutionary changes in just a few generations (Sriramulu et al. 2025; Barrett et al. 2008). The evolution of freshwater stickleback famously involves rapid loss of armour plating through fixation of low‐armour alleles at eda (Colosimo et al. 2005). This ecological transition, however, also involved changes in diet (which might impose selection on intestinal or liver cells involved in digestion), salinity (which might impact cells involved in osmoregulation). Immune evolution can also be important because during freshwater colonization, stickleback gained distinct communities of parasites (Bolnick et al. 2020a, 2020b).

Within a given cell type we can also identify the cell‐type‐specific genes that are evolving especially quickly (e.g., *pcolcea* in fibroblasts). Some noteworthy Cell‐TAGs exhibited particularly strong selection, even within cell types that otherwise were unremarkable on average. The strongest selection was observed for *cel* (a highly polymorphic gene in humans) within the acinar cell cluster (Figure 3a) (Taylor et al. 1991). This gene is involved in producing the enzyme carboxyl ester lipase, which is responsible for hydrolyzing dietary fat, esters and some vitamins and may be important in adaptation to changing nutrition during the transition from marine to freshwater habitat, as has been observed for the fatty acid desaturase gene *fads2 (*Johansson et al. 2018; Ishikawa et al. 2019). Other genes with strong selection in the intestine include ENSGACG00000010457 (currently undescribed) and *atp1a1a.2* (ATPase Na+/K+ transporting subunit alpha 1a, tandem duplicate 2). In the head kidney, we observed strong selection on *prdx5* in neutrophils, *pcolcea* in fibroblasts and *s100p* in antigen‐presenting cells (Figure 3b). *s100p* is present in mammals and teleosts, but is only partially represented in teleosts and was apparently lost in some teleost sublineages (Kraemer et al. 2008). This gene is differentially expressed in stickleback in response to parasite infection or antigen injection (Fuess et al. 2021).

Both empirical and theoretical evidence suggest that genetic interactions may influence the evolutionary rate of genes, for example, highly connected genes in a co‐expression network may be evolutionarily constrained due to their greater pleiotropy (Houle 2001; Fierst and Phillips 2012). This decoupling of microevolutionary rates (as measured by *F*_ST_) and macroevolutionary rates can have several causes. The most intriguing possibility is that frequency‐dependent selection imposed by host–parasite coevolution can lead to rapid evolutionary cycling of alleles (contributing to rapid among‐population divergence on short time scales), but long‐term stasis as polymorphism is retained. In single‐cell studies, network estimates are limited by sequencing depth/cell, though recent research suggests representing scRNAseq expression data as hypergraphs to handle varying levels of data sparsity and allow clustering algorithms to improve cell‐type differentiation (We et al. 2026).

This merger of scRNAseq and population genomic approaches thus provides a distinctive window into functional interpretation of evolutionary genomic data and lends further support to the insight that immune function is an especially labile trait over microevolutionary time. Additionally, this study demonstrates that scRNAseq may be used to help identify cell types within tissue samples and that these cell identities may be useful for inferring gene functions. Adding additional species and tissue types will continue improving our understanding of cell type identification, gene functions and rates of evolution across genes. This will also allow for identification of the ecological forces (e.g., parasite community differences) driving faster evolution in certain cell types. This perspective also poses an interesting possibility: as cell‐type‐associated genes (Cell‐TAGs) evolve, to what extent do cellular functions evolve as well? How constant are cell type identities and functions across microevolutionary divergence or macroevolutionary time?

## Author Contributions

The study was conceived and designed by D.I.B. and M.L.R. Single cell sequencing and analyses were performed by M.L.R., L.E.F. and M.S. Population genomic data collection and analyses were conducted by A.J.R.‐M. and S.S. Macroevolutionary rate analyses were conducted by D.L.J. Network analyses were conducted by W.H. and S.V.S. Statistical analyses and graphics were produced by D.I.B. and M.L.R. Funding acquisition was by D.I.B., K.M.‐M., N.C.S. and R.C. Initial draft was by M.L.R., D.I.B., W.H., D.L.J. and M.S. with comments and feedback from S.S., L.E.F., S.V.S., A.J.R.‐M., K.M.‐M., N.C.S. and R.C.

## Funding

This work was supported by the National Institute of Allergy and Infectious Diseases (1R01AI123659‐01A1), Gordon and Betty Moore Foundation (GBMF9323), and the National Institutes of Health (P30 CA034196).

## Ethics Statement

All fish were collected in accordance with the Ministry of Forests, Lands and Natural Resource Operations of British Columbia (Scientific Fish Collection permits NA12‐77018 and NA12‐84188). At the University of Connecticut, all fish were treated in accordance with an approved IACUC protocol (IACUC approval # A21‐025).

## Conflicts of Interest

The authors declare no conflicts of interest.

## Supporting information


**Figure S1:** A comparison of *F*_ST_ values in Alaska versus Vancouver Island for individual SNPs. There is a strong positive correlation between *F*_ST_ measured in the two regions (*r* = 0.538, *t* = 176.5, *p* < 0.0001) indicating a substantial element of parallel evolution. Overall, marine versus freshwater *F*_ST_ values were higher in Vancouver Island than in Alaska.
**Figure S2:** The frequency distribution of marine versus freshwater *F*_ST_ values for SNPs in (A) 1000 randomly selected genes (black) and in (B) the cell‐type specific genes (purple). These distributions have equivalent means (*F*_ST_ = 0.1463 and 0.1468, respectively, *t* = −0.839, *p* = 0.4013, df = 130,276), but slightly different distributions (KS‐test *D* = 0.0085, *p* = 0.0005) due to greater variance in the Cell‐TAGs' SNPs (0.0228 vs. 0.0215, *F*‐test *p* < 0.001).
**Figure S3:** The cell‐type specific genes of various cell populations differ in the strength of purifying selection over macroevolutionary time. We measure selection by calculating *dN*/*dS* ratios between stickleback species. *dN*/*dS* is the ratio of non‐synonymous to synonymous substitutions in coding sequences and is smaller for genes subject to stronger purifying selection that slows evolution. We present *dN*/*dS* for cell‐type specific genes, for each cell type within each of four tissues. With the exception of gills, all tissue types exhibit statistically significant differences in *dN*/*dS* between cell types.
**Figure S4:** Variation in allele frequency divergence (*F*_ST_) among cell‐type specific genes. Each cell‐type specific gene is represented along the *x* axis. Each SNP within each gene (including 5 kb of flanking sequence) is represented by a point, which is the mean *F*_ST_ between marine and freshwater populations for that given SNP. Averaging across SNPs within a gene yields the mean *F*_ST_ for an entire gene (blue points). Genes are ranked by mean *F*_ST_, so genes on the right exhibit the strongest signal of divergent selection.
**Figure S5:** Genic tree length (substitutions rate) for each of the four cell type clusters.

**Table S1:** Information for all 28 Pool‐seq populations used with Pool ID and Library ID, lake name, number of individuals, region of origin, sequencing centre and information on newly sequences versus previously sequences pools.
**Table S2:** Identified clusters for sequenced intestines with gene names and *p*‐values (*note:* only genes with *p*‐values of < 0.10 are represented for each cluster).
**Table S3:** Identified clusters for sequenced livers with gene names and *p*‐values (*note:* only genes with *p*‐values of < 0.10 are represented for each cluster).
**Table S4:** Identified clusters for sequenced gills with gene names and *p*‐values (*note:* only genes with *p*‐values of < 0.10 are represented for each cluster).
**Table S5:** Overall and regional values of gene‐level FST means, with standard deviations, ranges and the number of SNPs, for a sample of top‐FST genes.

## Data Availability

Poolseq population genomic data are archived on the SRA (accession numbers listed in Weber et al. 2022). Single‐cell RNA‐seq data are currently being archived on the Sequence Read Archive. R code to generate statistical results and graphics and associated intermediate data files (FST, *dN*/*dS*) with metadata are archived FigShare (https://doi.org/10.6084/m9.figshare.28452359).
