# Supplementary material for: Rates of Evolution Differ Between Cell Types Identified by Single‐Cell RNAseq in Threespine Stickleback
Source: Mol Ecol. 2026 Sep 28;35(19):e70565. doi: 10.1111/mec.70565 (PMC13619858; doi:10.1111/mec.70565)
Supplement: Supplementary file 1 [file MEC-35-e70565-s001.docx]

**Supplementary Figures**

**Figure S1.** A comparison of F_ST_ values in Alaska versus Vancouver Island for individual SNPs. There is a strong positive correlation between F_ST_ measured in the two regions (r = 0.538, t = 176.5, P < 0.0001) indicating a substantial element of parallel evolution.  Overall, marine versus freshwater F_ST_ values were higher in Vancouver Island than in Alaska.

**Figure S2.** The frequency distribution of marine versus freshwater F_ST_ values for SNPs in **A)** 1000 randomly selected genes (black), and in **B)** the cell-type specific genes (purple). These distributions have equivalent means (F_ST_ = 0.1463 and 0.1468 respectively, t = -0.839, P = 0.4013, df = 130,276), but slightly different distributions (KS-test D = 0.0085, P = 0.0005) due to greater variance in the Cell-TAGs’ SNPs (0.0228 vs 0.0215, F-test P < 0.001).

**Figure S3.** The cell-type specific genes of various cell populations differ in the strength of purifying selection over macroevolutionary time. We measure selection by calculating dN/dS ratios between stickleback species. dN/dS is the ratio of non-synonymous to synonymous substitutions in coding sequences, and is smaller for genes subject to stronger purifying selection that slows evolution. We present dN/dS for cell-type specific genes, for each cell type within each of four tissues. With the exception of gills, all tissue types exhibit statistically significant differences in dN/dS between cell types.

**Figure S4.** Variation in allele frequency divergence (F_ST_) among cell-type specific genes. Each cell-type specific gene is represented along the x axis. Each SNP within each gene (including 5kb of flanking sequence) is represented by a point, which is the mean F_ST_ between marine and freshwater populations for that given SNP.  Averaging across SNPs within a gene yields the mean F_ST_ for an entire gene (blue points). Genes are ranked by mean F_ST_, so genes on the right exhibit the strongest signal of divergent selection.

 

**Figure S5.** Genic tree length (substitutions rate) for each of the four cell type clusters.

Legends for Supplementary Tables.

**Table S1.** Information for all 28 Pool-seq populations used with Pool ID and Library ID, lake name, number of individuals, region of origin, sequencing center, and information on newly sequences vs previously sequences pools.

**Table S2.** Identified clusters for sequenced intestines with gene names and p-values (note: only genes with p values of <0.10 are represented for each cluster).

**Table S3.** Identified clusters for sequenced livers with gene names and p-values (note: only genes with p values of <0.10 are represented for each cluster).

**Table S4.** Identified clusters for sequenced gills with gene names and p-values (note: only genes with p values of <0.10 are represented for each cluster).

**Table S5.** Overall and regional values of gene-level FST means, with standard deviations, ranges, and the number of SNPs, for a sample of top-FST genes.
